# Synaptic Wiring of Corticostriatal Circuits in Basal Ganglia: Insights into the Pathogenesis of Neuropsychiatric Disorders

**DOI:** 10.1523/ENEURO.0076-19.2019

**Published:** 2019-06-04

**Authors:** Hsiao-Ying Kuo, Fu-Chin Liu

**Affiliations:** 1 Institute of Neuroscience, National Yang-Ming University, Taipei 11221, Taiwan; 2 Brain Research Center, National Yang-Ming University, Taipei 11221, Taiwan

**Keywords:** basal ganglia, corticostriatal circuits, neurodevelopmental diseases, striatum, synapse

## Abstract

The striatum is a key hub in the basal ganglia for processing neural information from the sensory, motor, and limbic cortices. The massive and diverse cortical inputs entering the striatum allow the basal ganglia to perform a repertoire of neurological functions ranging from basic level of motor control to high level of cognition. The heterogeneity of the corticostriatal circuits, however, also renders the system susceptible to a repertoire of neurological diseases. Clinical and animal model studies have indicated that defective development of the corticostriatal circuits is linked to various neuropsychiatric disorders, including attention-deficit hyperactivity disorder (ADHD), Tourette syndrome, obsessive-compulsive disorder (OCD), autism spectrum disorder (ASD), and schizophrenia. Importantly, many neuropsychiatric disease-risk genes have been found to form the molecular building blocks of the circuit wiring at the synaptic level. It is therefore imperative to understand how corticostriatal connectivity is established during development. Here, we review the construction during development of these corticostriatal circuits at the synaptic level, which should provide important insights into the pathogenesis of neuropsychiatric disorders related to the basal ganglia and help the development of appropriate therapies for these diseases.

## Significance Statement

Cortico-basal ganglia circuits control a range of neurobiological functions, ranging from motor control and reward to cognition. The functional diversity of cortico-basal ganglia circuits rests on their diverse inputs from the cerebral cortex. It is likely that the highly heterogeneous nature of corticostriatal inputs makes the corticostriatal circuits vulnerable to a broad range of neurologic and psychiatric disorders. Here, we highlight the developmental progression and maturation of the morphology and physiology of corticostriatal pathways using neonatal and postnatal rodent brains. We also review the pathogenesis of various neurodevelopmental disorders that are related to dysfunctions of corticostriatal circuits. Exploring synaptic wiring of corticostriatal circuits should create a research window for the development of therapeutic approaches for treating basal ganglia-related neurologic disorders.

## Introduction

An essential function of neural networks is the processing of sensory inputs and the generation of motor outputs. The cortico-basal ganglia circuitry is in a key hub that is involved in the integration of sensory and motor information by the brain. The striatum of the basal ganglia receives a large number of cortical inputs from the motor, sensory, association and limbic cortices (Hintiryan et al., 2016; Hunnicutt et al., 2016). The corticostriatal afferents thus make up a wide range input of different nature into the basal ganglia. The corticostriatal inputs are highly heterogeneous and therefore the cortico-basal ganglia circuits are involved in processing a broad spectrum of neurobiological functions. These range from motor control at a basic level to high level adaptive learning and cognition (Alexander et al., 1986; Graybiel, 2008; Pennartz et al., 2009; Haber, 2016). The integration of sensory and motor information is essential for behavioral performance and thus it is not surprising that dysfunction of cortico-basal ganglia circuits is well documented in a broad range of neurologic and mental disorders, including Parkinson's disease, Huntington's disease, attention-deficit hyperactivity disorder (ADHD), Tourette syndrome, obsessive-compulsive disorder (OCD), autism spectrum disorder (ASD), schizophrenia, and speech and language disorders (Crittenden and Graybiel, 2011; Shepherd, 2013; Gunaydin and Kreitzer, 2016).

**Figure 1.**
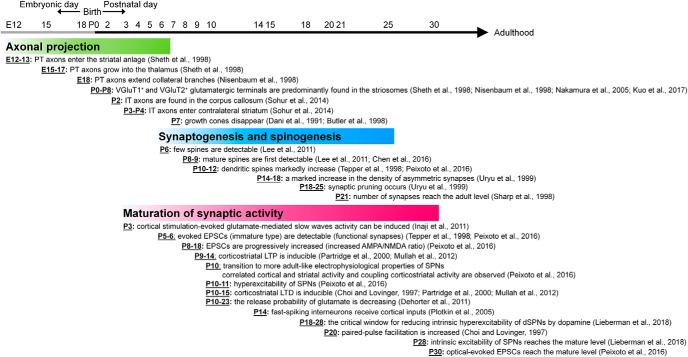
Developmental progression during the morphologic and physiologic maturation of corticostriatal innervations. AMPA: α-amino-3-hydroxy-5-methyl-4-isoxazolepropionic acid; dSPN; direct striatonigral pathway neuron; EPSC: excitatory postsynaptic current; iSPN: indirect striatopallidal pathway neuron; IT: intratelencephalic; LTD: long-term depression; LTP: long-term potentiation; NMDA: N-methyl-D-aspartate; PT: pyramidal tract; SPN: striatal projection neuron; VGluT1: vesicular glutamate transporter 1; VGluT2: vesicular glutamate transporter 2.

Many psychiatric disorders are rooted in developmental dysfunctions that affect neural circuits within the brain. Thus, it is imperative to understand how neural circuits are formed during development. In this review, we have focused on the construction at the cellular and molecular level during development of the corticostriatal circuits. We have summarized the developmental maturation timeline of corticostriatal innervations in Figure 1.

Genetic studies using mouse models have shown that many genes important to the development of neural circuits are involved in the pathogenesis of neurologic and psychiatric disorders. The significance and biological functions of these building blocks of neural circuits are, in fact, best illustrated by pathologic studies of neuropsychiatric diseases. We have therefore also reviewed the pathophysiology of neuropsychiatric disorders where it is known that dysfunction of corticostriatal circuits is involved. A summary of the various animal model studies that have shown links to corticostriatal abnormalities and neuropsychiatric diseases is presented in Table 1.

**Table 1.** Susceptible genes in neurodevelopmental diseases that are modeled in transgenic mice

Gene	Associated diseases	Morphological and functional phenotypes in corticostriatal circuits of transgenic mice carrying defective or variant alleles of neurodevelopmental disorder-risk genes	References
*Drd4*	ADHD	↓ corticostriatal glutamate release	Bonaventura et al. (2017)
*Shank3b*	ASDOCD	↑ dendritic arborizations↓ synaptogenesis/spinogenesis↑ precocious hyperactivity of corticostriatal inputs during development↓ corticostriatal synaptic transmission in adulthood	Peça et al. (2011) Peixoto et al. (2016)
*Fmr1*	ASD	↑ inhibitory neurotransmission in the striatum↓ corticostriatal plasticity (LTD)↓ corticostriatal connectivity	Centonze et al. (2008) Jung et al. (2012) Zerbi et al. (2018)
*Foxp1*	ASD	↑ excitability of iSPNs	Araujo et al. (2015)
*elf4e*	ASD	↑ spinogenesis in Layer II/III of medial prefrontal cortex↑ corticostriatal plasticity (LTD)	Santini et al. (2013)
*Nlgn1*	ASD	↓ NMDA-mediated synaptic transmission of dSPNs↓ mEPSCs in iSPNs	Blundell et al. (2010) Espinosa et al. (2015)
*Nlgn3*	ASD	↓ inhibitory synaptic transmission in dSPNs of the ventral striatum↓ corticostriatal plasticity (LTD)	Rothwell et al. (2014) Martella et al. (2018)
*Tshz3*	ASD	↑ corticostriatal LTP	Caubit et al. (2016)
*Met*	ASDSLD	↑ neuronal activity of corticostriatal pyramidal neurons in Layer Vb	Qiu et al. (2011)
*Mef2c*	ASDSLD	↑ synaptogenesis/spinogenesis in the striatum↑ mEPSCs in SPNs	Chen et al. (2016)
*Cntnap2*	ASDSLD	↓ number of striatal GABAergic interneurons	Peñagarikano et al. (2011)
*Slitrk5*	OCD	↓ BDNF-mediated neurite outgrowth of striatal neurons↑ neuronal activity of the orbitofrontal cortex↓ corticostriatal transmission↓ dendritic complexity of SPNs↓ GluR2, NR2B in the striatum	Song et al. (2015) Shmelkov et al. (2010)
*Sapap3*	OCD	↓ postsynaptic density thickness↑ NR1, NR2B and↑NR2A in the striatal PSD fraction↓ corticostriatal field EPSP↓ corticostriatal quantal EPSP in the iSPNs↓ corticostriatal feedforward inhibition of fast-spiking interneurons	Welch et al. (2007) Wan et al. (2014) Burguière et al. (2013)
*Slc1a1*	OCD	↓ neuronal activity in response to amphetamine in the dorsal striatum↓ NR2A and corticostriatal LTD in the dorsal striatum	Zike et al. (2017) Delgado-Acevedo et al. (2019)
*ErbB4*	Schizophrenia	↑ inhibitory synaptic transmission in the striatum↑ GABA_A_α1 in the striatum	Geng et al. (2017)
*Zswim6*	Schizophrenia	↓ neurite arborizations in the striatum↓ spinogenesis in the striatum	Tischfield et al. (2017)
*Foxp2*	SLD	↓ corticostriatal synaptic transmission↓ corticostriatal plasticity (LTD)↓ synaptogenesis/spinogenesis in the striatum↓ mEPSCs in SPNs	Groszer et al. (2008) Enard et al. (2009) Reimers-Kipping et al. (2011) Chen et al. (2016)

ADHD: attention-deficit hyperactivity disorder; ASD: autism spectrum disorder; BDNF: Brain-derived neurotrophic factor; dSPN: direct striatonigral pathway neuron; EPSC: excitatory postsynaptic current; EPSP: excitatory postsynaptic potential; GABA: γ-aminobutyric acid; GABA_Aalpha1_: GABA_A_ receptor subunit alpha 1; GluR2: glutamate ionotropic receptor AMPA type subunit 2; iSPN: indirect striatopallidal pathway neuron; LTD: long-term depression; LTP: long-term potentiation; NR1: glutamate ionotropic receptor NMDA type subunit 1; NR2A: glutamate ionotropic receptor NMDA type subunit 2A; NR2B: glutamate ionotropic receptor NMDA type subunit 2B; mEPSC: miniature excitatory postsynaptic currents; OCD: obsessive-compulsive disorder; PSD: postsynaptic density; SLD: speech and language disorders; SPN: striatal projection neurons.

## Establishment over Time of Corticostriatal Projections

Corticofugal pathways form the major afferent inputs to the basal ganglia. In the developing mouse brain, corticofugal projection neurons are classified into two cell types: pyramidal tract (PT)-type cortical projection neurons and intratelencephalic (IT) projection neurons (Reiner et al., 2003, 2010). Previous studies have revealed the developmental projections of the PT-type corticostriatal pathway. Nisenbaum et al. (1998) and Sheth et al. (1998) traced the developing corticofugal axons by placing DiI in fixed brain slices from a single hemisphere. The majority of the ipsilaterally DiI-labeled corticofugal axons are presumably derived from PT-type corticostriatal neurons, this is because the DiI-labeled axons can be traced and shown to exit the telencephalon to form part of the PT. However, because IT-type neurons project into both the ipsilateral and contralateral striatum, the possibility that some of the ipsilaterally DiI-labeled axons are derived from IT-type neurons cannot be excluded. Corticofugal axons have been found to enter the ipsilateral striatal anlage as early as embryonic day (E)12–E13. Corticofugal axons, presumably en route through the striatal analge during E12–E13, continue growing into the thalamus during E15–E17. By E18, clusters of collateral branches of corticofugal axons can be found in the ipsilateral striatum. Notably, these corticostriatal fibers are predominately co-localized with the tyrosine hydroxylase-positive nigrostriatal dopamine islands that are known to mark the loci of developing striosomes before the postnatal day (P)8 (Nisenbaum et al., 1998; Sheth et al., 1998). To explore the developmental projections of IT-type cortical neurons, Sohur et al. (2014) performed retrograde and anterograde labeling of corticostriatal axons during the first two postnatal weeks, and found that IT-type corticostriatal axons enter the contralateral striatum during P3–P4, and these corticostriatal axons progressively refined their innervation over the first two weeks after birth (Sohur et al., 2014). Immunostaining of growth-associated protein 43, a growth cone-specific protein, is markedly down-regulated in the dorsal striatum after P7, which suggests that there is a decrease in axonal growth in this region after P7 (Dani et al., 1991; Butler et al., 1998). Therefore, IT-type corticostriatal innervation of the mouse brain mainly starts during the early stage of the first postnatal week, and these innervations become mature by the second postnatal week.

## Topography of the Corticostriatal Pathways

Corticostriatal projections are topographically organized in such a manner as to convey neural information from various different cortical regions into the striatum where this diverse information is integrated. The dorsolateral/sensorimotor striatum, which is known to be involved in motor sequencing and habit-related functions, mainly receives its cortical inputs from the motor and somatosensory cortex. The dorsomedial/associative and ventral/limbic striata, which are related to goal-directed motor learning and reward, respectively, receive cortical inputs from the frontal cortex, mesocortex and allocortex (Alexander et al., 1986; McGeorge and Faull, 1989; Berendse et al., 1992; Mailly et al., 2013; Hintiryan et al., 2016; Hunnicutt et al., 2016). The deciphering of the topographic corticostriatal projection map has laid the foundations for understanding the functional networks related to a range of physiologic and pathologic conditions.

### Corticostriatal projections into striatal compartments

Considering the axonal terminal fields in the striatum, it has been shown that corticostriatal axons from different cortical regions innervate the striosomal and matrix compartments with different weights. The striosomal compartment receives substantial cortical inputs from evolutionarily conserved areas of the cortex, including the prelimbic, anterior cingulate, orbitofrontal, and insular cortices. The surrounding matrix compartment receives cortical inputs from the neocortex, including the motor, somatosensory and visual cortices (Gerfen, 1984; Donoghue and Herkenham, 1986; Ragsdale and Graybiel, 1990; Flaherty and Graybiel, 1993, 1994; Eblen and Graybiel, 1995; Kincaid and Wilson, 1996).

When the laminar distribution of the corticostriatal neurons is explored, the early-born PT-type corticostriatal projection neurons in the rat brain have been found to be primarily located in lower Layer V (Layer Vb), from where they project their axons into the striosomes. By way of contrast, the late-born IT-type corticostriatal neurons are located in Layer III and upper Layer V (Layer Va), from where they predominantly project their axons into the matrix compartment (Gerfen, 1989; McGeorge and Faull, 1989; Cowan and Wilson, 1994; Kincaid and Wilson, 1996; Lévesque and Parent, 1998). Interestingly, a recent genetic study using virus-based axonal tracing has revealed that there is no preference regarding corticostriatal afferents entering into the striatal compartments of the mouse brain (Smith et al., 2016). Cre-dependent monosynaptic tracing via genetically modified rabies virus using compartment-specific Cre driver mice by Smith et al. (2016) has found that both the patch/exo-patch (striosome) compartment and the matrix compartment receive cortical innervations from the limbic and sensorimotor cortices without any preference. Cortical neurons in the upper and deep layers were shown to project into both the patch/exo-patch (striosome) compartment and the matrix compartment (Smith et al., 2016). Nonetheless, single-cell RNA sequencing has identified genetically distinct cell populations in a single cortical area (Tasic et al., 2016). It is possible that different populations of cortical neurons within a single cortical region may project differentially into the striatal compartments. A combination of the single-cell RNA sequencing linked with axonal tracing and behavioral studies may help to clarify the above issue.

### Corticostriatal projections linking to distinct striatofugal pathways

Evidence based on retrograde axonal tracing and electron microscopy have shown that more corticostriatal axons of the IT-type compared to the PT-type are linked to neurons of the direct striatonigral pathway (dSPNs). In contrast, more PT-type than IT-type corticostriatal axons are linked to neurons of the indirect striatopallidal pathway (iSPNs; Lei et al., 2004; Reiner et al., 2010; Deng et al., 2015). When the efferent regions of the cortex are explored, it remains unclear as to whether there is differential innervation of dSPNs and iSPNs from distinct cortical regions (Berretta et al., 1997; Wall et al., 2013; Guo et al., 2015). Wall et al. (2013) have reported that axons from the sensory and limbic cortices preferentially innervate dSPNs, whereas axons from the motor cortex preferentially project toward iSPNs (Wall et al., 2013). However, Guo et al. (2015) did not find that there was differential innervation of dSPNs and iSPNs by the various different cortical regions. This inconsistency may be the result of variation in the degree of virus infection present in a single cortical region across the two studies. Alternatively, it also may be due to differences in trans-synaptic retrograde efficiency across the various different types of corticostriatal synapses (Guo et al., 2015).

## Corticostriatal Axonal Outgrowth and Synaptogenesis

A coculture study has demonstrated the specificity of corticostriatal innervation by prenatal and postnatal cortical afferents. During cortical and striatal coculture, neurites derived from the prenatal cortex homogeneously grow into co-cultured striatal tissue, while, on the other hand, neurites derived from perinatal stages specifically grow into the striosomal compartment of co-cultured striatum irrespective of the age of the striatal tissue. Only a small amount of neurite innervations can be found in striatal tissue when it is co-cultured with postnatal cortex (Snyder-Keller, 2004). These results suggest a time-dependent interplay between the different cortical afferents and the striatum. Because corticostriatal axonal innervation occurs before synaptogenesis and functional connectivity is established during the postnatal period (see below), corticostriatal axonal innervation during the various embryonic stages may occur without much in the way of activity modulation from the cortex.

Corticostriatal synaptogenesis occurs postnatally after corticostriatal axonal innervation has been established. A cell culture study has shown that cortical synaptic inputs into the striatum are important for maturation of the dendritic arborization of the SPNs (Buren et al., 2016). SPNs receives glutamatergic excitatory inputs from VGluT1-positive corticostriatal neurons and VGluT2-positive thalamostriatal neurons (Fremeau et al., 2001, 2004). Corticostriatal and thalamostriatal axonal terminals form axospinous and axodendritic synapses with the SPNs, respectively (Dubé et al., 1988). The synaptogenesis of the excitatory synapses occurs soon after axonal innervations during postnatal periods. Interestingly, VGluT1 and VGluT2 immunoreactivity are highly enriched in striosomal loci during the first postnatal week, suggesting that corticostriatal and thalamostriatal synapse formation start to occur in the neonatal striosomal compartment (Nakamura et al., 2005; Kuo and Liu, 2017). The dendritic spines of SPNs are found low in the P6 striatum, and mature types of dendritic spines are first able to be detected during the period P8–P9 (Lee and Sawatari, 2011). Dendritic spines markedly increase in number during the period P10–P12, at which time the SPNs are at their most excitable during the postnatal period. Dendritic spines continue to increase until the fourth postnatal week (Tepper et al., 1998; Chen et al., 2016; Peixoto et al., 2016). The numbers of asymmetric synapses found in the P21 striatum at this time are comparable to those found in the adult rat striatum (Sharpe and Tepper, 1998). Considering the synaptic pruning, there is a dramatic decrease in dendritic spines that occurs within the dorsolateral rat striatum, but not the dorsomedial rat striatum from P18 to P25 (Uryu et al., 1999). It should also be noted that synaptic pruning by microglia has also been documented in the postnatal brain (Paolicelli et al., 2011; Wu et al., 2015).

During the early postnatal stages, developmental maturation of the electrophysiological properties of the SPNs involves immature SPNs that are characterized by a depolarized resting membrane potential together with hyperexcitability and a lack of inward rectification (Tepper et al., 1998; Peixoto et al., 2016). When developmental maturation of corticostriatal innervations of SPNs occurs, this happens in parallel concomitant with developmental corticostriatal innervations and dendritic spinogenesis, while at the same time cortical stimulation-evoked glutamate-mediated slow wave activity is able to be induced in the SPNs of P3 brain slices as shown by a voltage-sensitive dye (Inaji et al., 2011). By P5–P6, electrical-evoked and optical-evoked EPSCs are able to be detected in SPNs, suggesting the presence of functional corticostriatal synapses at this stage (Tepper et al., 1998; Peixoto et al., 2016). Optical-evoked EPSCs progressively increase with the gradual recruitment and stabilization of AMPA receptors during the period P8–P18. Optical-evoked EPSCs reach a mature level by P30 (Peixoto et al., 2016). Interestingly, a decrease in the release probability of glutamate during the development of the corticostriatal synapses has been observed during striatal long-term depression (LTD) over the period P10–P23, which correlates with the motor function developmental maturation. Moreover, it has been reported that a loss of NMDA NR2C/D-mediated corticostriatal inputs occurs concurrently with a decrease in an immature pattern of striatal activity before P10 and that this is correlated with the onset of locomotion by the neonatal mouse pups (Dehorter et al., 2011).

Cortical synaptic inputs not only drive synaptic activity in SPNs, but are also able to indirectly inhibit SPN activity via fast-spiking interneuron-mediated feed-forward inhibition (Plotkin et al., 2005). Electrophysiological recordings of fast-spiking interneurons in the striatum of rat brain slices obtained during the periods P12–P14 and P19–P23 have shown that fast-spiking interneurons have received frequent cortical inputs by the end of the second week after birth, which is when corticostriatal synaptogenesis is ongoing. This suggests a potential role for fast-spiking interneuron-mediated cortical feed-forward inhibition during the development of corticostriatal circuits (Plotkin et al., 2005).

In the dorsolateral striatum, corticostriatal long-term potentiation (LTP) is inducible as early as P9–P10, whereas LTD cannot be induced until P15 (Choi and Lovinger, 1997; Partridge et al., 2000; Mullah et al., 2012). This developmental transition affecting the synaptic plasticity of LTD is controlled by upregulation of the endogenous cannabinoid ligand anandamide (Ade and Lovinger, 2007). The release probability of glutamate from cortical axonal terminals has been shown to decrease from P10 to P23, which may account for the induction of striatal LTD. LTD is known to be a physiologic mechanism that underlies motor performance, learning, and memory. Taken together, the above findings delineate the functional maturation process during postnatal development of corticostriatal synaptogenesis.

## Neuronal Activity-Dependent Regulation of Corticostriatal Synaptogenesis during Development

### Glutamate inputs

A previous study has suggested that recurrent activity in the closed loops of cortico-basal ganglia circuits is able to regulate the synaptogenesis of SPNs (Kozorovitskiy et al., 2012). Synaptic connectivity and the strength of corticostriatal pathways are subject to regulation by the outputs from basal ganglia circuits. Chronic inhibition of activity within dSPNs and iSPNs during the second postnatal week by chemogenetic manipulation has been shown to result in decreased and increased spinogenesis and miniature EPSCs (mEPSCs) in dSPNs and iSPNs, respectively (Kozorovitskiy et al., 2012). Acute and chronic elevation of cortical activity by the inactivation of cortical interneurons or the optogenetic activation of corticostriatal axonal terminals has been shown to increase the synaptic connectivity of the corticostriatal pathways during early development (Peixoto et al., 2016). Moreover, correlated increases in cortical and striatal activity has been observed during the period P10–P16 (Peixoto et al., 2016). These findings suggest that corticostriatal inputs are capable of modulating activity-dependent synaptogenesis in the SPNs.

### Dopamine inputs

Dopaminergic inputs into basal ganglia circuits have been suggested to regulate corticostriatal maturation. Mesostriatal dopaminergic afferents start to innervate the striatum during early embryonic stages. At the perinatal stage, mesostriatal dopaminergic axonal terminals form “dopamine islands” that correspond to the developing striosomes (Olson et al., 1972; Graybiel, 1984; van der Kooy, 1984). During the period P8–P13, activation of Gα_s_-coupled G-protein receptors in SPNs by a D1 agonist is able to increase corticostriatal activity and the number of dendritic spines (Kozorovitskiy et al., 2015). Furthermore, depletion of dopamine input into the P2 striatum weakens not only SPN activity in response to cortical stimulation, but also impairs the corticostriatal synchronization that accompanies locomotion induced hyperactivity during the period P21–P25 (Galiñanes et al., 2009).

Moreover, the imbalances in dopamine D1 and D2 signaling in SPNs that occur before the first two postnatal weeks has been shown to lead to significant alterations in corticostriatal innervation and spinogenesis (Kozorovitskiy et al., 2012). A recent study has further shown that nigrostriatal dopamine release during a critical period between P18 and P28 is required to reduce the intrinsic hyperexcitability of neonatal dSPNs to the level found in adults (Lieberman et al., 2018). Taken together, the above compelling evidence highlights the importance of dopamine transmission, not only in the regulation of the postnatal maturation of SPNs, but also it associated with the pathogenic mechanisms related to neurodevelopmental disorders.

### Brain-derived neurotrophic factor (BDNF)

BDNF is one of the most studied neurotrophins and has been shown to be involved in the pathogenesis of various neurodevelopmental and neuropsychiatric disorders (Autry and Monteggia, 2012; Park and Poo, 2013). BDNF binds to tyrosine receptor kinase B (TrkB) to transduce signals within developing neurons. Evidence from BDNF and TrkB conditional knock-out mice has indicated that BDNF-TrkB signaling regulates the neuronal survival, morphogenesis and synaptogenesis of striatal neurons (Baydyuk et al., 2011; Li et al., 2012); furthermore, presynaptic BDNF secretion is required for activity-dependent corticostriatal LTP (Park et al., 2014). Notably, TrkB expression is enriched in the striosomal compartment during the first postnatal week when corticostriatal axons are selectively innervating striosomal cells (Costantini et al., 1999). These findings raise an intriguing possibility that BDNF-TrkB signaling may be involved in setting up within the striatal compartments the temporal order during activity-dependent corticostriatal synaptogenesis; this could involve initiating the initial steps of synapse formation within the striosomal compartment.

## Neuropsychiatric Diseases Related to Dysfunction of the Corticostriatal Circuits

### Attention-deficit hyperactivity disorder (ADHD)

ADHD is a neurodevelopmental disease that is characterized by symptoms including inattention and/or hyperactive/impulsive behaviors that persist for more than six months (American Psychiatric Association, 2013). Based on neuroimaging and genetic studies, abnormal neural connectivity and abnormal neurologic functioning are believed to underlie the pathology of ADHD brains (Mueller et al., 2017). Aberrant neural circuits, including dorsal frontostriatal, orbitofrontostriatal, prefrontostriatal, and frontoparietal circuits, have been identified as being associated with ADHD (Durston et al., 2011; Hong et al., 2015; Mueller et al., 2017).

Many ADHD studies have centred on dysfunction of the dopaminergic system (Levy, 1991). Dopamine neurotransmission is essential for motor control, reward learning, and motivation (Björklund and Dunnett, 2007; Gerfen and Surmeier, 2011). In addition to regulating neurotransmission within the adult brain, dopamine also plays an important role in neural development. Notably, depletion of dopamine by intraventricular injection of 6-hydroxydopamine (6-OHDA) into neonate rodent brains has been shown to induce an ADHD-like behavioral phenotype; furthermore, abnormal development of corticostriatal pathways and synaptogenesis has been found in 6-OHDA-treated brains (Galiñanes et al., 2009; Braz et al., 2015). Moreover, altered frontostriatal functional connectivity, which has linked to a reduction in the dendritic arborizations of SPNs, in particular iSPNs, has been also observed in juvenile 6-OHDA-treated mice. These neonatal 6-OHDA-induced pathologic changes become more severe in adult mice compared to juvenile mice (Braz et al., 2015). Transgenic knock-in mice expressing a human DRD4 polymorphic variant associated with ADHD have been shown to exhibit a decrease in corticostriatal glutamate release (Bonaventura et al., 2017). These different lines of evidence suggest that the corticostriatal development controlled by dopaminergic transmission may underlie ADHD pathophysiology.

### Autism spectrum disorder (ASD)

ASD is a highly heterogeneous disease. The core symptoms of ASD include impaired social communication functioning and self-interest related repetitive behaviors (American Psychiatric Association, 2013; Wingate et al., 2014). Aberrant synaptogenesis, plasticity and excitatory/inhibitory balance are believed to be involved in ASD pathophysiology (Toro et al., 2010; Bourgeron, 2015; Nelson and Valakh, 2015). The complex yet specific symptoms of ASD pathogenesis are presumably caused by aberrant wiring of specific neural circuits in the ASD brain. Evidence suggests that dysfunction of the basal ganglia circuits plays an important role in ASD pathogenesis and is related to repetitive behavior and defective social communication (Portmann et al., 2014; Ellegood et al., 2015; Fuccillo, 2016). Clinical studies have reported that there is an increase in the volume of the striatum that is positively correlated with the repetitive behaviors, the social deficits and the communicational deficits of the patients (Hollander et al., 2005; Rojas et al., 2006). Neuroimaging studies have shown a correlation between inward surface deformation affecting distinct striatal regions and impaired motor skills, praxis and poorer social communication (Qiu et al., 2010). Moreover, reduced long-range functional connectivity between the right inferior frontal cortex and the right caudate has been observed in brain of children suffering from ASD (Lee et al., 2009). These findings imply that there are dysfunctions that affect the corticostriatal and striatofugal circuits of ASD brains. Many genes that have been associated with ASD have been found to be related to corticostriatal development and synaptogenesis, including the *Shank* gene family and the *Neuroligin* (NLGN) gene family. *Shank1*, *Shank2*, and *Shank3* are members of a postsynaptic scaffolding SH3 and multiple ankyrin repeat domains protein family. Mutations of the *Shank* family proteins are associated with autism and a number of other neuropsychiatric disorders (Phelan, 2008; Leblond et al., 2014; Monteiro and Feng, 2017). Mutation of the ASD-risk gene *Shank3B,* which is the most well-studied isoform, has been found to be enriched in corticostriatal regions, and to be associated with an increase in dendritic arborizations and a decrease in dendritic spinogenesis and synaptogenesis of SPNs (Peça et al., 2011). Mutation of *Shank3B* also leads to precocious hyperactivity of corticostriatal inputs due to the presence of cortical hyperactivity during P14; while, in contrast, lower levels of corticostriatal connectivity have been found in adult brains with this mutation (Peça et al., 2011; Peixoto et al., 2016).

Mutations affecting the NLGN gene family induce corticostriatal synaptopathy and this seems to be related to the ASD pathogenesis (Sudhof, 2008). For example, *NLGN3* mutant mice exhibit increased inhibitory synaptic transmission in dSPNs of the ventral striatum and impaired corticostriatal LTD in the dorsal striatum (Rothwell et al., 2014; Martella et al., 2018). *NLGN1,* another NLGN member that has recently been identified as an ASD-risk gene (Nakanishi et al., 2017), has been shown to regulate NMDA-mediated synaptic transmission in dSPNs and mEPSC frequency in iSPNs (Blundell et al., 2010; Espinosa et al., 2015).

In addition to genes in the Shank and NLGN families, other ASD risk genes also seem to alter corticostriatal connectivity and activity. Human transcriptome analysis has identified Teashirt zinc-finger homeobox family member 3 (*TSHZ3*) as a hub gene that is involved in the development of cortical projection neurons. Patients with deletion mutation of the *TSHZ3* gene exhibit ASD symptoms, and an animal model study has shown that there is an increase in corticostriatal LTP in *Tshz3* heterozygous mice (Caubit et al., 2016). Furthermore, *Fmr1* encodes the fragile X mental retardation protein that is involved in autistic Fragile X syndrome. In *Fmr1* knock-out mice, an increase in inhibitory neurotransmission and defects in LTD have been found in the striatum and this has been linked with hypoconnectivity of the corticostriatal pathways (Centonze et al., 2008; Jung et al., 2012; Zerbi et al., 2018). Eukaryotic translation initiation factor 4E (*eIF4E*) is another gene associated with ASD susceptibility (Neves-Pereira et al., 2009). Overexpression of *eIF4E* in transgenic mice results in increased dendritic spines in Layers II and III of the medial prefrontal cortex as well as enhanced striatal LTD (Santini et al., 2013). Mutation of transcription factor forkhead box p1 (*Foxp1*), an ASD-risk gene, is known to increase the neuronal excitability of iSPNs during P18 (Araujo et al., 2015). Finally, a reduction in putative cortico-striosomal synaptogenesis has been found in the P8 striatum of a ASD mouse model involving maternal treatment of mice with valproic acid (Kuo and Liu, 2017). Collectively, the above animal model studies suggest that dysfunction of corticostriatal synaptic homeostasis and disruption of synaptogenesis during development may contribute to the pathologic mechanisms of ASD.

### Obsessive-compulsive disorder (OCD)

OCD is a neurodevelopmental disease wherein dysfunction of corticostriatal circuits at the neural circuit level is implicated in the pathology of the disease. Neuroimaging studies have found abnormalities of corticostriatal pathways, including the orbitofrontal cortex, prefrontal cortex, anterior cingulate cortex and striatum in the brains of patients with OCD (Harrison et al., 2009; Rotge et al., 2009; Anticevic et al., 2014; Hou et al., 2014; Jung et al., 2017). The pathologic alterations in these corticostriatal circuits have been correlated with the severity of the patient’s OCD symptoms (Harrison et al., 2013). Moreover, repetitive transcranial magnetic stimulation and deep-brain stimulation-induced changes in corticostriatal activity seem to be able to cause alterations in the symptoms in OCD patients (Figee et al., 2013; Dunlop et al., 2016), which supports a causal relationship between dysfunction of corticostriatal circuits and OCD pathogenesis (Burguière et al., 2015; Fettes et al., 2017). Animal model studies have shown that chronically optogenetic activation of the orbitofronto-ventral striatum pathways is sufficient to bring about a progressive increase in the obsessive grooming behavior of mice, and that fluoxetine, a clinical drug used to treat OCD, is able to alleviate the optogenetic-induced obsessive grooming behaviors (Ahmari et al., 2013).

At the genetic level, mutations of several genes have been associated with OCD. It is notable that corticostriatal circuitry appears to be a convergent pathologic locus that is targeted by several OCD-risk genes, these include synapse-associated protein 90/postsynaptic density protein 95-associated protein 3 (*SAPAP3*) and members of the *Slitrk* gene family. SAPAP3 is an excitatory postsynaptic scaffold protein. Mutations of *SAPAP3* have been found in patients with OCD (Züchner et al., 2009). *Sapap3* knock-out mice exhibit defects affecting the structure of the postsynaptic complex and show a OCD-like behavioral phenotype. Moreover, the synaptic activity of the corticostriatal pathways, but not the thalamostriatal pathways, are altered, which indicates that the corticostriatal pathways seem to be specifically relevant to OCD pathophysiology (Welch et al., 2007; Wan et al., 2014). Compulsive grooming, an abnormality associated with *Sapap3* knock-out mice, is able to be rescued by optogenetic activation of parvalbumin-positive fast-spiking interneuron-mediated feed-forward inhibition of the orbitofrontal-striatal circuits (Burguière et al., 2013). The *Slitrk* gene family is known to regulate synaptogenesis (Takahashi et al., 2012; Yim et al., 2013). Mutations of *Slitrk* gene family members are associated with various neuropsychiatric diseases, including Tourette syndrome and OCD (Abelson et al., 2005; Ozomaro et al., 2013). Slitrk5 is known to regulate the recruitment of TrkB into the postsynaptic regions to bring about BDNF-mediated neurite outgrowth of striatal neurons (Song et al., 2015). Genetic deletion of *Slitrk5* not only results in neuronal hyperactivity in the orbitofrontal cortex, but also causes a reduction in corticostriatal transmission and dendritic complexity that is associated with OCD-like behaviors (Shmelkov et al., 2010).

In addition to mutation of the *SAPA3* and *Slitrk5* genes, mutations of the *SLC1A1* and *Hoxb8* genes have been shown to be related to abnormalities in corticostriatal activity and to changes in corticostriatal connectivity that have in turn been associated with OCD phenotypes. The *SLC1A1* gene encodes neuronal glutamate/aspartate/cysteine transporter excitatory amino acid transporter 3 (EAAT3). An abnormal increase in *SLC1A1* expression with a parallel decrease in EAAT3 activity, have been found in the brains of patients with OCD (Veenstra-VanderWeele et al., 2001; Dickel et al., 2006; Wendland et al., 2009). EAAT3-deficient mice exhibit diminished basal ganglia-dependent stereotypic behavior (Zike et al., 2017). A recent study has further reported that NMDA receptor subunit composition and NMDA-dependent synaptic plasticity are altered in mice overexpressing EAAT3 and this is linked to OCD-like behavioral deficits; these deficits can be rescued by antipsychotic treatment (Delgado-Acevedo et al., 2019). Interestingly, Hoxb8 function in microglia has been suggested to be involved in the pathogenesis of OCD via regulation of the corticostriatal circuits. *Hoxb8* gene knock-out has been shown to induce an expansion in cortical synapses and contraction in striatal synapses. These changes then lead to an enhancement of corticostriatal activity in the *Hoxb8* mutant mice, which can be seen to exhibit compulsive grooming behavior (Greer and Capecchi, 2002; Nagarajan et al., 2018).

### Schizophrenia

Schizophrenia is a neurodevelopmental disease. Imbalanced excitatory/inhibitory transmission and abnormal synaptic function have been implicated in the pathophysiology of schizophrenia. Genetic linkage studies have indicated that the *AKT1* and *PRODH* genes are associated with schizophrenia (Albert et al., 2002; Kempf et al., 2008; Thiselton et al., 2008). Elevated frontostriatal connectivity, particular in the dorsolateral prefrontal cortex, is present in subjects carrying AKT1 and PRODH variant alleles (Meyer-Lindenberg et al., 2007; Kempf et al., 2008; Tan et al., 2008). Corticostriatal dysfunction affecting cognitive learning and reward processing have been identified among patients with schizophrenia (Wagshal et al., 2014; de Leeuw et al., 2015). In animal studies, NRG1-ErbB4 signaling (Jaaro-Peled et al., 2009) has been proposed to underlie the pathogenesis of schizophrenia. Conditional deletion of ErbB4 in Dlx5/6 cell lineage cells, including SPNs and cortical interneurons, results in enhanced inhibitory synaptic transmission in the striatum (Geng et al., 2017). Loss of zinc-ﬁnger SWIM domain-containing protein 6 (*ZSWIM6*), another schizophrenia-risk gene, has been shown to decrease neurite arborization and the number of dendritic spines in SPNs (Tischfield et al., 2017). Abnormal dopamine transmission has been a prevailing theory for the etiology of schizophrenia (Simpson et al., 2010). In this context, it is interesting to note that mesostriatal dopamine input during development is able to modulate corticostriatal innervations, spinogenesis and activity during development (Galiñanes et al., 2009; Kozorovitskiy et al., 2012; Lieberman et al., 2018). These findings suggest that abnormal synaptic wiring within the corticostriatal circuits may be a potential pathologic mechanism that underlies abnormal dopamine neurotransmission in schizophrenia brains.

### Speech and language disorders

Speech and language are unique and fundamental to human beings and their social communication. The prevalence rate for language delay during the development of school children is high, which calls for a better understanding of the neurobiology of speech and language and the development of new therapeutic approaches to such problems. Cortico-basal ganglia circuits are important to speech and language (Watkins et al., 1999; Dick et al., 2014; Graham and Fisher, 2015; Konopka and Roberts, 2016). Several genes related to speech and language have been identified using genome-wide linkage/association sequencing studies (Newbury and Monaco, 2010; Kang and Drayna, 2011; Graham and Fisher, 2015; Konopka and Roberts, 2016). A well-studied speech and language-related gene is *FOXP2*. The *FOXP2* R553H missense mutation caused severe level of speech and language disorder in the KE family (Lai et al., 2001). Neuroimaging studies have found structural abnormalities as well as functional abnormalities that affect the cerebral cortex and striatum of patients with a *FOXP2* mutation. This suggests there is involvement of cortico-basal ganglia circuits in the pathology of this disorder (Watkins et al., 1999; Liégeois et al., 2003). Consistent with the above, animal studies have indicated that the cortico-basal ganglia circuits are critical to vocal communication in songbirds and rodents (Enard et al., 2009; Fisher and Scharff, 2009; Arriaga et al., 2012; Condro and White, 2014; Konopka and Roberts, 2016). Increasing evidence indicates that *Foxp2* plays a crucial role in the neural development and plasticity of corticostriatal circuits. Mutation of the *Foxp2* gene decreases synaptic transmission and plasticity in corticostriatal synapses (Groszer et al., 2008; Enard et al., 2009; Reimers-Kipping et al., 2011). Humanized *Foxp2* gene has been shown to increase synapse formation and synaptic functioning in the SPNs from mouse brains (Enard et al., 2009; Chen et al., 2016). It is important to note that developmental deficits in spoken language function are a comorbidity associated with various other psychiatric disorders, including ASD in which vocal communication can also be severely affected. Interestingly, Foxp2 has been shown to negatively regulate several ASD-risk genes, including *Cntnap2*, *Met*, and *Mef2c* (Vernes et al., 2008; Mukamel et al., 2011; Chen et al., 2016). Our recent study has uncovered a molecular mechanism by which Foxp2 promotes the synaptogenesis of corticostriatal circuits via suppression of Mef2c, a negative regulator of synapse formation. The Foxp2-Mef2c signaling-mediated synaptic wiring of corticostriatal circuits has a causative role in vocal communication (Chen et al., 2016). Cntnap2 may modulate corticostriatal inputs via regulation of the development of striatal GABAergic interneurons (Peñagarikano et al., 2011). Finally, *Met* knock-out mice have been shown to exhibit hyperactivity of corticostriatal pyramidal neurons in cortical Layer Vb, which may affect the development of corticostriatal pathways (Qiu et al., 2011).

Cortico-basal ganglia circuits engage with other neural circuits to control complex neurobiological functions such as speech and language. The corticostriatal circuits may be a good entry point by which one may explore the developmental basis of vocal communication and this should be able to provide insights into the pathogenesis of various speech and language disorders.

### Tourette syndrome

Tourette syndrome is a childhood-onset neurodevelopmental disease that is characterized by involuntary vocal and motor tics. Tourette syndrome is categorized as one of the three tic disorders in DSM-V (American Psychiatric Association, 2013). Tourette syndrome is a heterogeneous disorder that shows comorbidity with other neuropsychiatric diseases, including OCD and ADHD (Hirschtritt et al., 2015). The pathophysiology of Tourette syndrome is poorly understood. At the circuit level, increasing evidence had implicated pathologic changes in cortico-striato-pallido-thalamic circuits within the brains of patients with Tourette syndrome (McNaught and Mink, 2011). Neuroimaging studies have found abnormal increases in structures associated with corticostriatal connectivity, and these are further correlated with the severity of the tics (Govindan et al., 2010; Worbe et al., 2015), At the genetic level, twins siblings studies of individuals with Tourette syndrome have indicated that the concordance rate of monozygotic twins is higher than that of dizygotic twins (McNaught and Mink, 2011). Furthermore, genetic studies have identified a number of candidate genes that are associated with Tourette syndrome. One possible candidate gene is the Slit and Trk-like 1 (*SLITRK1*) gene. Patients carrying *SLITRK1* variant alleles have known to exhibit Tourette syndrome (Abelson et al., 2005). Slitrk1 is expressed at high levels in cortical Layers III, V, and VI, which is where the corticostriatal and corticothalamic projection neurons are located in the mouse brain. Slitrk1 is also highly expressed in postnatal striosomes. Expression of slitrk1 has been shown to be significantly down-regulated after the second postnatal week, which is when corticostriatal innervation and synaptogenesis occur. The expression pattern of *SLITRK1* in the cerebral cortex and striatum of the human brain is similar to that found in the monkey brain and the mouse brain (Stillman et al., 2009). Previous studies have shown that Slitrk1 positively regulates dendrites outgrowth and synaptogenesis in cortical pyramidal neurons and hippocampal neurons (Abelson et al., 2005; Yim et al., 2013; Beaubien et al., 2016). Given that Slitrk1 is expressed during the development of corticostriatal pathways, a potential role for Slitrk1 in the regulation of corticostriatal connectivity and its possible involvement in Tourette syndrome warrants further investigation.

## Conclusion

A broad repertoire of genetic components participates in the synaptic wiring of the corticostriatal circuits during development. Activity-dependent machinery is also adopted to fine tune synaptogenesis to develop precise functionality within the cortico-basal ganglia network. Characterization of the development and functioning of corticostriatal circuits may not only help us to understand the developmental basis of motor control, skill development and habit learning as well as complex cognitive functions such as speech and language, but may also provide insights into the pathophysiology of corticostriatal circuits-related neurologic and psychiatric disorders. This, in turn, may lead to the identification of potential therapeutic approaches to the treatment of these important diseases.

## Synthesis

Reviewing Editor: Juan Burrone, King's College London Decisions are customarily a result of the Reviewing Editor and the peer reviewers coming together and discussing their recommendations until a consensus is reached. When revisions are invited, a fact-based synthesis statement explaining their decision and outlining what is needed to prepare a revision will be listed below. The following reviewer(s) agreed to reveal their identity: NONE.

This review provides a comprehensive and detailed description of the wiring of cortico-striatal circuits in health and disease. The review was well organised and well discussed. It is a welcome summary of the large body of work that exists in this area. Overall, I find that this is an important and timely review.

However, there were some minor issues that the authors should address. For example, the review contained multiple sentences that were either not clear or grammatically incorrect. The authors should take time to revisit the review in detail and iron out these issues. Below are specific comments on the review.

The introduction has many sentences that need rewriting. Below are some examples, but the authors should carefully read through this section and modify so that it reads more clearly.

Delete ‘the’ form first line of introduction to read: ‘are critical to network function’.

Second sentence (starting with ‘The cortico-basal ganglia...’) is not clear.

Fourth sentence of introduction (starting with ‘The cortico-striatal afferents...’) is also not clear.

13th line of introduction: insert ‘be’ so that sentence reads ‘...to be involved in a broad...’

Second paragraph of introduction, first sentence: sentence does not read well.

Second paragraph of introduction, second sentence: change to ‘at the cellular and molecular level’

Second paragraph of introduction, third sentence does not read well.

Page 4, 5th line: insert ‘The’ to start sentence ‘The majority...’

Page 4, 2nd paragraph, first sentence, change to: ‘...Sohur et al (2014) performed retrograde and anterograde labelling of...’

Page 4, 2nd paragraph, last sentence: what do you mean by establish the innervations?

Page 6 second paragraph: sentence starting ‘Previous studies...’ is confusing.

Page 7, line 3: start sentence with ‘A previous cell culture...’

Page 7: what does Asymmetric synaptogenesis mean? Is this the synaptogenesis of asymmetric synapses? If so, please define asymmetric synapses or refer to them as excitatory synapses.

Page 8, second paragraph, line 2: switch to ‘interneuron-mediated’.

Page 8, second paragraph, line 5: change to ‘by the end of the second week’.

Page 8, second paragraph, line 6: change ‘are undergoing’ with ‘ is ongoing’.

Page 8, last paragraph, line 1: start with ‘Previous studies’

Page 9, first paragraph: sentence starting with ‘Moreover, correlated increases in cortical...’ is unclear.

Page 10: In ADHD section, what is meant by ‘physiologic function’?

Page 11, last paragraph: This sentence does not make much sense, as the genes listed have not been mentioned before.

Page 12: this whole section on genes related to ASD may benefit from a common thread that links genes together. As it stands it reads like a long list of genes involved in ASD but it is not always clear why the review jumps for one gene to another.

Pages 13-14: a similar comment applies to genes involved in OCD.
